# Supplementary material for: The Impact of Muscarinic Antagonism on Psychosis-Relevant Behaviors and Striatal [11C] Raclopride Binding in Tau Mouse Models of Alzheimer’s Disease
Source: Biomedicines. 2023 Jul 25;11(8):2091. doi: 10.3390/biomedicines11082091 (PMC10452133; doi:10.3390/biomedicines11082091)
Supplement: Supplementary file 1 [file biomedicines-11-02091-s001.zip › Compressed ZIP/Supplemental Table 1.docx]

| Gene Name |  | log2FoldChange | Wald test  P-value | Benjamini-Hochberg adjusted P-value |
| --- | --- | --- | --- | --- |
| ACHE | **Acetylcholinesterase** | 0.003655 | 0.97 | NS |
| CHAT | **Choline O-Acetyltransferase** | 0.1955 | 0.36 | NS |
| CHRM1 | **Cholinergic Receptor Muscarinic 1** | -0.2597 | ***9.9E-04*** | NS |
| CHRM2 | **Cholinergic Receptor Muscarinic 2** | 0.08286 | 0.60 | NS |
| CHRM3 | **Cholinergic Receptor Muscarinic 3** | -0.1268 | 0.15 | NS |
| CHRM4 | **Cholinergic Receptor Muscarinic 4** | -0.3766 | ***1.7E-05*** | ***0.0097*** |
| CHRM5 | **Cholinergic Receptor Muscarinic 5** | 0.1594 | 0.57 | NS |
| TH | **Tyrosine Hydroxylase** | -0.1876 | 0.61 | NS |
| DDC | **Dopa Decarboxylase** | 0.3961 | ***0.015*** | NS |
| COMT | **Catechol-o-Methyltransferase** | -0.7107 | 0.74 | NS |
| MAOA | **Monoamine Oxidase A** | -0.0100 | 0.93 | NS |
| MAOB | **Monoamine Oxidase B** | 0.0403 | 0.67 | NS |
| DRD1 | **Dopamine Receptor D1** | 0.0594 | 0.99 | NS |
| DRD2 | **Dopamine Receptor D2** | -0.0337 | 0.68 | NS |
| DRD3 | **Dopamine Receptor D3** | 1.375 | ***0.047*** | NS |
| DRD4 | **Dopamine Receptor D4** | -0.7758 | 0.83 | NS |
| DRD5 | **Dopamine Receptor D5** | 0.3676 | 0.44 | NS |

Supplemental Table 1. RNA-Seq log_2_-fold-change (LFC) estimates of gene expression in the muscarinic cholinergic and dopamine pathways in scopolamine versus saline treated P301L/COMTKO mice. Negative LFC values represent relatively under-expressed genes, positive LFC values represent relatively over-expressed genes.
