# Supplementary material for: The Impact of Muscarinic Antagonism on Psychosis-Relevant Behaviors and Striatal [11C] Raclopride Binding in Tau Mouse Models of Alzheimer’s Disease
Source: Biomedicines. 2023 Jul 25;11(8):2091. doi: 10.3390/biomedicines11082091 (PMC10452133; doi:10.3390/biomedicines11082091)
Supplement: Supplementary file 1 [file biomedicines-11-02091-s001.zip › Compressed ZIP/Supplemental Table 2.docx]

| **Filenames** | ***2011_10_30_MRI_template3** | ****ROI-23-CPU**  **(75367 voxels***)** | ****ROI-32-NAC**  **(10990 voxels***)** | ***ROI-35-CER** |
| --- | --- | --- | --- | --- |
| 20220324_a655sal_FDG_s1_WcXdcbb | 1.60E+01 | 1.71 | 1.63 | 1.95E+01 |
| 20220329_a665sal_FDG_s1_WcXdcbb | 8.44E+02 | 1.74 | 1.55 | 1.01E+03 |
| 20220408_a645sal_FDG_s1_WcXdcbb | 1.84E-02 | 1.68 | 1.56 | 2.23E-02 |
| 20220412_a648sal_FDG_s1_WcXdcbb | 1.50E+01 | 1.43 | 1.34 | 2.06E+01 |
| 20220412_a668sal_FDG_s1_WcXdcbb | 1.63E+01 | 1.64 | 1.55 | 2.00E+01 |
| 20220614_a751sal_FDG_s1_WcXdcbb | 5.81E-09 | 1.58 | 1.49 | 7.41E-09 |
| 20220617_a752sal_FDG_s1_WcXdcbb | 9.88E-09 | 1.60 | 1.52 | 1.26E-08 |
| 20220621_a753sal_FDG_s1_WcXdcbb | 9.04E-09 | 1.60 | 1.37 | 1.13E-08 |
| 20220623_a759sal_FDG_s1_WcXdcbb | 8.77E-09 | 1.56 | 1.39 | 1.10E-08 |
|  |  |  |  |  |
| 20220324_a656scop_FDG_s1_WcXdcbb | 1.48E+01 | 1.70 | 1.68 | 1.76E+01 |
| 20220329_a666scop_FDG_s1_WcXdcbb | 8.93E+02 | 1.63 | 1.52 | 1.11E+03 |
| 20220408_a646scop_FDG_s1_WcXdcbb | 1.85E-02 | 1.50 | 1.27 | 2.52E-02 |
| 20220412_a657scop_FDG_s1_WcXdcbb | 2.23E+01 | 1.54 | 1.41 | 2.89E+01 |
| 20220412_a669scop_FDG_s1_WcXdcbb | 2.09E+00 | 1.56 | 1.42 | 2.70E+00 |
| 20220614_a794scop_FDG_s1_WcXdcbb | 7.55E-09 | 1.59 | 1.54 | 9.65E-09 |
| 20220621_a756scop_FDG_s1_WcXdcbb | 7.26E-09 | 1.73 | 1.61 | 8.80E-09 |
| 20220623_a757scop_FDG_s1_WcXdcbb | 8.55E-09 | 1.58 | 1.48 | 1.07E-08 |
|  |  |  |  |  |
| AVE-Saline | 99.08 | 1.62 | 1.49 | 119.07 |
| STD-Saline | 279.61 | 0.09 | 0.10 | 334.78 |
| AVE-Scopolamine | 116.47 | 1.60 | 1.49 | 145.17 |
| STD-Scopolamine | 313.69 | 0.08 | 0.13 | 390.88 |
| p-value-SalinevsScopolamine | 0.91 | 0.74 | 0.97 | 0.88 |

Supplemental Table 2 ^18^FDGPET:
No significant difference in FDG-PET between saline and scopolamine mice was found in caudate-putamen (dorsal striatum) and nucleus accumbens (ventral striatum) (p > 0.74, two tailed Student’s t-test).

*Raw value

**Normalized by cerebellum value

***1 voxel = 0.07x0.07x0.07 mm^3^
