# Supplementary material for: The Impact of Muscarinic Antagonism on Psychosis-Relevant Behaviors and Striatal [11C] Raclopride Binding in Tau Mouse Models of Alzheimer’s Disease
Source: Biomedicines. 2023 Jul 25;11(8):2091. doi: 10.3390/biomedicines11082091 (PMC10452133; doi:10.3390/biomedicines11082091)
Supplement: Supplementary file 1 [file biomedicines-11-02091-s001.zip › Compressed ZIP/Supplemental Table 3.docx]

| **Filenames** | ***2011_10_30_MRI_template3** | ****ROI-23-CPU**  **(75367 voxels***)** | ****ROI-32-NAC**  **(10990 voxels***)** | ***ROI-35-CER** |
| --- | --- | --- | --- | --- |
| 20220324_a655Sal_11Crac_s1_WcXdcbb | 1.16E+01 | 2.69 | 2.28 | 1.15E+01 |
| 20220329_a665sal_11Crac_s1_WcXdcbb | 1.45E+01 | 2.16 | 1.89 | 1.58E+01 |
| 20220408_a645sal_11Crac_s1_WcXdcbb | 6.31E+00 | 2.63 | 2.19 | 6.36E+00 |
| 20220412_a648Sal_11Crac_s1_WcXdcbb | 9.44E+00 | 2.60 | 2.28 | 9.18E+00 |
| 20220412_a668Sal_11Crac_s1_WcXdcbb | 4.83E+00 | 2.35 | 1.92 | 5.16E+00 |
| 20220614_a751sal_11Crac_s1_WcXdcbb | 2.56E-09 | 2.45 | 2.07 | 2.80E-09 |
| 20220617_a752sal_11Crac_s1_WcXdcbb | 3.24E-09 | 2.39 | 1.99 | 3.38E-09 |
| 20220621_a753sal_11Crac_s1_WcXdcbb | 4.82E-13 | 2.11 | 1.76 | 5.46E-13 |
| 20220623_a759sal_11Crac_s1_WcXdcbb | 2.56E-09 | 2.40 | 2.01 | 2.81E-09 |
|  |  |  |  |  |
| 20220324_a656scop_11Crac_s1_WcXdcbb | 1.15E+01 | 2.46 | 2.02 | 1.22E+01 |
| 20220329_a666scop_11Crac_s1_WcXdcbb | 1.07E+01 | 2.26 | 1.89 | 1.20E+01 |
| 20220408_a646scop_11Crac_s1_WcXdcbb | 9.00E+00 | 2.21 | 1.81 | 9.93E+00 |
| 20220412_a657scop_11Crac_s1_WcXdcbb | 1.26E+01 | 2.23 | 1.89 | 1.35E+01 |
| 20220412_a669scop_11Crac_s1_WcXdcbb | 7.09E+00 | 2.23 | 1.92 | 7.59E+00 |
| 20220614_a794scop_11Crac_s1_XWcdcbb | 2.98E-09 | 2.37 | 2.06 | 3.17E-09 |
| 20220621_a756scop_11Crac_s1_WcXdcbb | 4.54E-13 | 2.08 | 1.71 | 5.06E-13 |
| 20220623_a757scop_11Crac_s1_WcXdcbb | 2.97E-09 | 2.22 | 1.91 | 3.26E-09 |
|  |  |  |  |  |
| AVE-Saline | 5.19 | 2.42 | 2.04 | 5.33 |
| STD-Saline | 5.65 | 0.20 | 0.18 | 5.88 |
| AVE-Scopolamine | 6.36 | 2.26 | 1.90 | 6.90 |
| STD-Scopolamine | 5.51 | 0.11 | 0.11 | 5.98 |
| p-value-SalinevsScopolamine | 0.67 | 0.06 | 0.07 | 0.59 |

Supplemental Table 3. There was a decrease in ^11^C-Raclopride in scopolamine mice (n=8) relative to saline mice (n=9) in caudate-putamen (dorsal striatum) and nucleus accumbens (ventral striatum) ( P = 0.035, one-tailed Student’s t-tests )

*Raw value

**Normalized by cerebellum value

***1 voxel = 0.07x0.07x0.07 mm^3^
