# Supplementary material for: The Impact of Muscarinic Antagonism on Psychosis-Relevant Behaviors and Striatal [11C] Raclopride Binding in Tau Mouse Models of Alzheimer’s Disease
Source: Biomedicines. 2023 Jul 25;11(8):2091. doi: 10.3390/biomedicines11082091 (PMC10452133; doi:10.3390/biomedicines11082091)
Supplement: Supplementary file 1 [file biomedicines-11-02091-s001.zip › Compressed ZIP/Supplemental Table 4.docx]

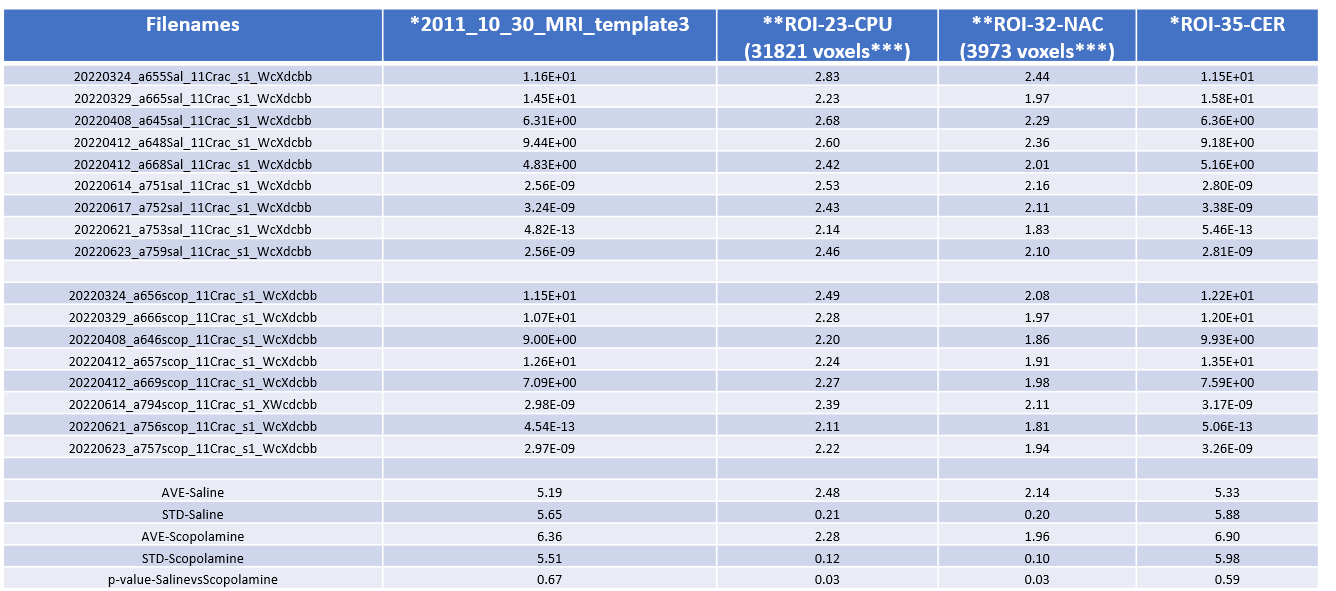


Supplemental Table 4 There was a significant decrease in ^11^C-Raclopride in scopolamine mice (n=8) relative to saline mice (n=9) in caudate-putamen (dorsal striatum) and nucleus accumbens (ventral striatum) when a mask obtained from SPM analysis with ^11^C-Raclopride in Scopolamine < in Saline at p < 0.01 was applied

*Raw value

**Normalized by cerebellum value

***1 voxel = 0.07x0.07x0.07 mm^3^
